# Dendritic Cell Vaccination of Glioblastoma: Road to Success or Dead End

**DOI:** 10.3389/fimmu.2021.770390

**Published:** 2021-11-02

**Authors:** Angeliki Datsi, Rüdiger V. Sorg

**Affiliations:** Institute for Transplantation Diagnostics and Cell Therapeutics, Heinrich-Heine University Hospital, Medical Faculty, Düsseldorf, Germany

**Keywords:** dendritic cells, vaccination, immunotherapy, glioblastoma, review (article), glioma, brain tumor

## Abstract

Glioblastomas (GBM) are the most frequent and aggressive malignant primary brain tumor and remains a therapeutic challenge: even after multimodal therapy, median survival of patients is only 15 months. Dendritic cell vaccination (DCV) is an active immunotherapy that aims at inducing an antitumoral immune response. Numerous DCV trials have been performed, vaccinating hundreds of GBM patients and confirming feasibility and safety. Many of these studies reported induction of an antitumoral immune response and indicated improved survival after DCV. However, two controlled randomized trials failed to detect a survival benefit. This raises the question of whether the promising concept of DCV may not hold true or whether we are not yet realizing the full potential of this therapeutic approach. Here, we discuss the results of recent vaccination trials, relevant parameters of the vaccines themselves and of their application, and possible synergies between DCV and other therapeutic approaches targeting the immunosuppressive microenvironment of GBM.

## Introduction

Glioblastomas (GBM) are highly invasive, malignant tumors of the central nervous system. According to the 2021 World Health Organization classification, GBM are grade 4 tumors, that belong to the group of adult diffuse gliomas (1). They lack mutations in the isocitrate-dehydrogenase (IDH) gene, which now discriminates GBM from IDH-mutated grade 4 astrocytomas, which have been regarded as secondary GBM before. Based on gene expression signatures, GBM can be further subdivided into mesenchymal, proneural, neural and classical subtypes (2).

Although representing the most frequent malignant primary brain tumor (~30%–40%), GBM are rare; the yearly incidence is three to four per 100,000 adults (3). Nevertheless, they are a highly fatal tumor, responsible for 2% of cancer-related deaths, with a yearly death rate of four to five per 100,000 adults. The established therapeutic standard of care in the first-line therapy for GBM combines maximal safe resection, fractionated radiotherapy with concomitant alkylating temozolomide (TMZ) chemotherapy, followed by adjuvant TMZ treatment. This multimodal approach has improved survival of patients significantly. Nevertheless, prognosis of newly diagnosed GBM patients is dismal. Median overall survival (mOS) is only 14.6 months, and the 2-year survival rate is 27.2% (4, 5). In GBM patients with unmethylated *O* ^6^-methylguanine-DNA-methyltransferase (MGMT) promoter, producing the DNA-repair enzyme, prognosis is even worse [methylated *vs.* unmethylated mOS is 21.7 *vs.* 12.7 months; (6)]. Disease recurrence is universal, there is no effective therapy for recurrent disease, and median survival after relapse is 6.2 months. Therapeutic alternatives include lomustine, carmustine, tumor-treating field (TTF) therapy, and the angiogenesis inhibitor bevacizumab (7–10). Thus, there is a clear need for novel therapeutic modalities in GBM.

Dendritic cells (DC) are professional antigen-presenting cells, which are key to the development of T-cell responses (11, 12). As immature (resting) cells, they reside in most tissues, where they sample antigens. When activated by pathological changes in the tissue, they migrate to the draining lymph nodes and present there as mature (activated) DC peptides processed from the antigenic material taken up in the tissue on human leukocyte antigen (HLA) class I and II molecules, in an immunostimulatory context of co-stimulatory and accessory molecules. Antigen-specific cytotoxic T lymphocyte (CTL) and helper T cells (T_H_) recognizing these peptides get activated, proliferate, and differentiate to effector cells, which execute the various actions of cellular adaptive immune responses, including the killing of target cells. DC vaccination (DCV) is an active immunotherapy seeking to exploit this pivotal role of DC therapeutically: patients are vaccinated with tumor-associated antigens (TAA)-loaded DC, with the concept that they migrate to local lymph nodes, present TAA-derived peptides on HLA molecules, and initiate an antitumoral T-cell response, which selectively kills the tumor cells and prevents tumor recurrence, due to immunological memory (13, 14).

DCV was first evaluated in 1996 in a clinical trial for B-cell lymphoma (15). In 1999, Dhodapkar et al. reported the induction of antigenic target-directed T-cell responses by DCV (16). The authors vaccinated nine healthy individuals with mature DC loaded with an influenza matrix peptide, keyhole limpet hemocyanin, or tetanus toxoid and showed induction of target-specific T-cell immunity after a single application of the vaccine. The clinical efficacy of DCV was documented in 2006 in a phase III trial in patients with hormone refractory prostate cancer (17). Patients were treated with either placebo (leukocytes) or DC loaded with a fusion protein of prostatic acid phosphatase and granulocyte-macrophage colony-stimulating factor (GM-CSF) (Provenge/sipuleucel-T). mOS of vaccinated patients was significantly improved compared with that in the placebo-treated control group (25.9 *vs.* 21.4 months), results that were confirmed in a second trial (18); and Provenge/sipuleucel-T was the first (and so far only) DC vaccine approved by the U.S. Food and Drug Administration in 2010 (19). In GBM, hundreds of patients have been vaccinated, mainly in smaller uncontrolled trials. Although the results are promising, few studies can provide robust evidence, and overall the efficacy of DCV in GBM is variable, ranging from little or no clinical response to significant response. Therefore, we address the questions of which parameters could possibly effect efficacy of DCV and whether and how it may be possible to improve it.

## Dendritic Cell Vaccination for Glioblastoma in Animal Models

Already in 1999, Liau and colleagues documented in the 9L rat glioma model that vaccination with DC pulsed with acid-eluted peptides derived from 9L glioma cells can prolong survival of glioma-bearing animals (20). In addition, vaccination was associated with infiltration of tumors with CD8^+^ and, to a lesser extent, CD4^+^ T cells and the development of glioma 9L cell-specific CTL responses. In 2000, Heimberger et al., who had vaccinated mice with DC pulsed with lysates derived from the spontaneously arising 560 glioma cell line, which had been transfected with the murine homolog of the mutated epidermal growth factor receptor variant III (EGFRvIII) (21), reported that vaccination could protect mice from subsequent intracranial tumor challenge. Survival of vaccinated animals was significantly prolonged compared with that in control animals receiving unpulsed DC. The surviving animals showed antitumoral memory and were healthy, were neurologically normal, and showed no signs of autoimmune encephalitis. Again, vaccination was associated with the development of glioma-cell-specific CTL responses, but interestingly, those were not directed against EGFRvIII, but against other unknown TAA.

Meanwhile, numerous animal studies have been performed in prophylactic (22–24) and curative DCV settings (25–31). Overall, there is clear evidence from these initial animal studies that DCV reduces tumor growth, can prolong survival, induces tumor-specific IFNγ and CTL responses, is associated with T-cell infiltration of tumors, particularly by CD8^+^ T cells, and results in long-lasting antitumoral memory that provides protection from tumor re-challenge. At the same time, vaccination appears to be safe and not to be associated with the development of autoimmunity. Thus, animal studies provided a proof-of-principle for DCV of GBM. Moreover, they continue to contribute to the development of vaccination strategies to increase efficacy (32).

## Treatment of Glioblastoma Patients With Dendritic Cell Vaccination

Active immunotherapy with DCV has been pioneered by Liau et al., who described the vaccination of a GBM patient with recurrent disease with DC pulsed with eluted peptides of an HLA class I-matched GBM cell culture in 2000 (33). Although induction of an anti-peptide immune response was observed, the patient progressed and died 3 months later. Since then, numerous studies have been published (**Table 1**), including six controlled, randomized trials (60, 62, 76, 79, 81, 83), and several more are underway (88). Patients included mainly adults, but also children and adolescents (34, 38, 39, 41, 48, 52, 61, 64, 65, 69, 78), and the age of vaccinated patients varied between 1 and 80 years.

**Table 1:** Concluded clinical trials and case reports on dendritic cell vaccination of glioblastoma patients.

Diagnosis All/nd GBM/rec GBM control	Antigenic target	^7^DC maturation	DC application	DC dose number vaccines/cells/vaccine	Clinical outcome (GBM) OS/PFS/others	Immunological responses (GBM) DTH IFNg^10^ others^11^	Toxicity^9^	Ref
rec GBM 1/0/1	Eluted peptides	-	i.d.	3× biweekly 5 × 10^6^	PD	- - 1	None	(33)
rec HGG 8/0/5	HGG/DC fusion	TNFα	i.d.	1–8× triweekly 2.4–8.8 × 10^6^	1× MR, 1× SD	- 5/5 -	Erythema	(34)
nd HGG 9/7/0	Eluted peptides	-	s.c.	3× biweekly 1 × 10^6^	nd: 15.0 m/–	- - 4/7^12^	Fever, lymph node swelling, vomiting/nausea	(35)
nd/rec GBM 42/24/18	Tumor lysate	–	s.c.	3× biweekly ± 1× after 6 weeks 10–40 × 10^6^				(36)
rec HGG 10/0/7	Tumor lysate	-	i.d. + i.t.	1–10× triweekly 10–32 × 10^6^	rec^8^: 17.7 m/– 4× SD	3/6 2/4 -	Headache, erythema	(37)
rec HGG 9/0/2	Tumor mRNA	–	i.d. + i.v.	3× biweekly ± 3× monthly 5 × 10^6^/m^2^ (i.v.) + 5 × 10^6^ (i.d.)	PD	- 0/4^12^ 0/3^12^	None	(38)
rec GBM 1/0/1	Tumor lysate	TNFα, IL-1β, PGE2	i.d.	2× biweekly + 4× monthly 1–9 × 10^6^	CR (2 years)		None	(39)
rec HGG 15/0/6	HGG/DC fusion	TNFα	i.d.	3× biweekly 3.6–32.3 × 10^6^	rec^8^: 8.5 m/– 1× SD (4 m)	- 0/6 0/8	Fever, seizure, erythema, transient liver dysfunction, lymphopenia	(40)
rec HGG 12/0/7	Tumor lysate	TNFα, IL-1β, PGE2	i.d.	2× biweekly + 4× monthly 0.8–18 × 10^6^	1× CR	6/8^12^ - -	Peritumoral edema (grade 4), morning stiffness, hematotoxicity, nocturnal sweating, meningeal irritation	(41)
nd/rec GBM 25/11/14	Tumor lysate/ eluted peptides	–	s.c.	3× biweekly ± 1× after 6 weeks 10–40 × 10^6^	nd: 34.4 m/– rec: 29.6 m/–	- 40%–60%^12^ 60%^9^		(42)
nd/rec HGG 14/1/9	Tumor lysate	-	s.c.	3× biweekly 10–100 × 10^6^	rec: 30.6 m/–	- nd: 0/1; rec: 4/5 nd: 0/1; rec: 2/6	Headache, fatigue, erythema, seizure	(43)
nd/rec GBM 12/6/6	Eluted peptides	–	i.d.	3× biweekly 1, 5 or 10 × 10^6^	nd^8^: 27.9 m/16.3 m rec^8^: 16.6 m/12.5 m	- - nd: 4/6; rec: 2/6	Fever, flu-like, fatigue, myalgia, nausea/vomiting, erythema, itching, lymph node swelling, diarrhea/constipation	(44)
rec HGG 24/0/18	Tumor lysate	- or OK432	i.d. + i.t.	1–22× (i.d.) 0-18× (i.t.) triweekly 1–32 × 10^6^	rec^8^: 15.5 m/– 2× MR, 2× SD	8/15 6/13 -	Headache, erythema	(45)
rec GBM 1/0/1	Tumor lysate	–	i.v.	5× biweekly			Fever	(46)
nd GBM 6/6/0	Tumor lysate	TNFα, IL-1β, IFNγ	i.d.	2× biweekly 2 × 10^6^	nd^8^: –/6.0 m	- 0/5 -	Headache	(47)
rec GBM 56/0/56	Tumor lysate	TNFα, IL-1β, PGE2	i.d.	3–7×: 2 biweekly + others monthly or 3–9× biweekly or 4× weekly 0.7–25.7 × 10^6^	rec: 9.6 m/3.0 m	9/17 - -	Peritumoral edema (grade 4), hematotoxicity, hemiparesis, dysphasia, headache, vomiting, flu-like, seizure, fatigue, myalgia, hygroma, intratumoral hemorrhage, erythema	(48)
nd/rec HGG 13/7/2	Irradiated tumor cells	MCM	i.d.	2–13×: 6× biweekly + every 6 weeks 1 × 10^6^	nd^8^: 11.0 m/– rec^8^: 5.0 m/–		None related to DCV	(49)
nd/rec HGG 44/11/23	Tumor lysate	–	s.c.	3× biweekly + 1× after 6 weeks 10–40 × 10^6^		- 17/34^12^ -	No grade 3/4	(50)
nd GBM 12/12/0	EGFRIII peptide-KLH conjugate	TNFα, IL-1β, IL-6	i.d.	3× biweekly 30–100 × 10^6^	nd: 22.8 m/10.2 m	5/9 - 10/12	Increased erythrocyte sedimentation rate, increased rheumatoid factor level	(51)
rec HGG 45/0/23	Tumor lysate	TNFα, IL-1β ± PGE2	i.d.	3–7×: 2 biweekly + others monthly or 4–16× biweekly or 4× weekly 0.5–23.8 × 10^6^	rec^8^: 12.2 m/4.3 m		Fatigue, headache, fever, itching, vomiting, flu-like	(52)
nd GBM 8/8/0	Tumor lysate	TNFα, IL-1β, PGE2	i.d.	4× weekly 2–24 × 10^6^	nd: 24.0 m/18.0 m	2/7 5/8 -	Lymphopenia, focal epileptic insult, dysphasia, fatigue, malaise, myalgia, ischemic event (grade 4), hematotoxicity (grade 3), status epilepticus (grade 4)	(53)
nd/rec HGG 17/8/6	Heat-shocked irradiated cells	-	s.c.	4× weekly + 2× biweekly + 4× monthly 10–60 × 10^6^	nd^8^: 12.1 m/– rec^8^: 31.8 m/–		Lymphopenia (grade 3/4), transient hepatic dysfunction, seizure, hydrocephalus, anemia, myalgia, skull wound infection	(54)
nd GBM 10/10/0	Tumor lysate	TNFα, PGE2	i.n.	3× biweekly 30 × 10^6^	nd: 28.0 m/9.5 m	0/10 4/10 -	Neck pain	(55)
rec HGG 22/0/13	Peptides^1^	TNFα, IL-1β, IFNα, IFNγ, poly(IC)	i.n.	4× biweekly + 5× monthly 10 or 30 × 10^6^	rec^8^: 12.0 m/4.0 m 1× CR (>13 m), 1× PR	- 5/12 5/10	Erythema, flu-like, fatigue, myalgia, fever, chill/rigor, headache, lymphopenia	(56)
nd/rec GBM 23/15/8	Tumor lysate	–	i.d.	3× biweekly ± ≤10× 3-monthly 1 or 5 × 10^6^	nd: 35.9 m/– rec: 17.9 m/–		Fatigue, nausea/vomiting, diarrhea, arthralgia, fever, lymphadenopathy, erythema, myalgia, shingles, allergic rhinitis, pruritus, headache, constipation, heartburn, dermatitis/rash, anorexia, abdominal pain	(57)
rec HGG 9/0/7	Peptides^2^	TNFα, IL-1β, IFNα, IFNγ, poly(IC)	i.d.	4× weekly ± ≤ 6× 10, 20, or 50 × 10^6^	PD	4/7 4/6 -	Transient hepatic dysfunction	(58)
nd GBM 77/77/0	Tumor lysate	TNFα, IL-1β, PGE2	i.d.	4× weekly 0.24–55 × 10^6^	nd: 18.3 m/10.4 m		Fatigue, rash/itching, shoulder pain, anorexia, myalgia, nausea/vomiting, seizure, confusion, humerus fracture, lethargy, ectopic cerebral lesion, depression, dysphasia, esophagitis, otitis media serosa, lymphopenia, leukopenia; grade 3/4 seizure, allergic reaction to TMZ, cerebral abscess, deep vein thrombosis, hydrocephalus, ischemic bowel perforation, lung and peripheral edema, osteoporotic fracture, dementia, focal status epilepticus, ischemic stroke, status epilepticus, thrombocytopenia, lymphopenia, leukopenia; grade 5: overwhelming infection	(59)
nd GBM 18/18/0 16	Tumor lysate	-	s.c.	4× weekly + 2× biweekly + 4× monthly 20-50 × 10^6^	nd: 31.5 m/8.5 m		Hepatic dysfunction, lymphopenia, hemiplegia, pancytopenia, intracranial pressure, nausea/vomiting	(60)
rec HGG 8/0/5	IL-13Rα2 peptides	TNFα, IL-1β, IL-6	i.d.	2–6× biweekly 10 × 10^7^	rec^8^: 7.0 m/– 1× MR, 2× SD (2-4 m)	- 2/3 2/3	Fatigue, erythema	(61)
nd GBM 13/13/0 12	Tumor lysate	TNFα, IL-1β, PGE2	s.c.	2× weekly + 2× biweekly 6 × 10^6^	nd: 17.0 m/11.9 m 3× CR (89 m), 6× PR, 1× SD (9 m)	- Increase -	Fever, erythema	(62)
nd GBM 5/5/0	Tumor lysate	TNFα, IFNα, poly(IC)	?	4–14: 2× bimonthly + 2× monthly + 4× bimonthly + others quarterly 0.8–10 × 10^6^	nd: 27.4 m/16.1 m	- - 3/3	Seizure	(63)
nd HGG 7/5/0	Tumor lysate	-	i.d.	2–4 biweekly 1 × 10^6^		- - -	Headache, injection site erythema, elevated alkaline phosphatase (grade 4)	(64)
rec GBM 15/0/15	Tumor lysate	TNFα IL-1β IL-6 PGE2	i.d.	3–4 × biweekly 2× monthly 1/2 × 10^7^	rec: 8 m/4.4 m	- Increase NK cell response	Ependymitis/hydrocephalus, anemia grade 2, fever, cutaneous induration, cutaneous flushing, seizures, cerebral edema, tumor bleeding	(65)
nd/rec GBM 19/16/3	Peptides^3^	TNFα	i.d.	3× biweekly 10 × 10^6^	nd: 38.4 m/16.9 m	- 5/15^12^ 5/15^12^	Diarrhea, fatigue, flushing, pruritus, rash, vomiting	(66)
nd/rec HGG 27/23/4	Tumor lysate/peptides	–/ TNFα, IL-1β, IL-6, PGE2	i.d.	3× biweekly/3× biweekly + 3× monthly	nd 34.4 m/18.1 m rec: 14.5 m/9.6 m	- - Increase	Grade 1–2 flu-like (headache, low-grade fever, nausea, vomiting, fatigue), injection site reactions, lymphadenopathy, rashes	(67)
nd GBM 7/7/0	GBM CSC mRNA	TNFα, IL-1β, IL-6, PGE2	i.d.	9–18×: 2×/week + 3× weekly + others monthly 10 × 10^6^	nd: 25.0 m/22.8 m	1/7 - 7/7	Fatigue, anorexia, nausea, seizure, constipation, fatigue (grade 3)	(68)
rec HGG 8/0/6	Apoptotic bodies allogeneic CSC GBM6-AD	TNFα, IL-1β, IFNα, IFNγ, poly(IC)	s.c.	1–9×: 5× biweekly + 5× monthly	3× SD	- 0/6^13^ 3/6^13^	Fatigue, erythema, induration (grade 1)	(69)
rec GBM 14/0/14	Irradiated tumor cells	MCM	i.d.	3× biweekly 6× monthly 4 × 10^6^/ 1 × 10^6^	rec^8^: 23.0 m/5.0 m 2× PR, 1× SD (31.5 m)	0/14 2/9 -	Nausea/vomiting, headache, seizure, thrombocytopenia, syncopal event (grade 3), bilateral cataracts (grade 3)	(70)
nd GBM 13/13/0	CMVpp65 mRNA	TNFα, IL-1β, IL-6, PGE2	i.d.	3× biweekly + others monthly 20 × 10^6^	nd: 18.5 m/10.8 m	- Durable -	None related to vaccine	(71)
rec HGG 10/0/6	WT-1 peptides/ tumor lysate	OK432, PGE2	i.d.	5–7× biweekly + ≤23× 7–99.4 × 10^6^	rec: 18.0 m/– 1× SD	5/5 - 3/4	Erythema, fever, fatigue	(72)
nd/rec GBM 32/22/10	GBM/DC fusion	TNFα	i.d.	3× monthly + 6/12-monthly 0.72–2.5 × 10^6^	nd: 30.5 m/18.3 m rec: 18 m/10.3 m	- Positive 4/4	Injection site reaction	(73)
nd GBM 11/11/0	CMVpp65 mRNA	TNFα, IL-1β, IL-6, PGE2	i.d.	10× biweekly and monthly 20 × 10^6^	41.1 m/25.3 m	- 10/11 Increase	No AE related to DCV except for GM-CSF autoantibody (grade 3)	(74)
nd GBM 32/32/0	Tumor lysate	TNFα, IFNα, poly(IC)	i.d.	>6 10 × 10^6^	23.4 m/12.7 m	- 8/25 No change	No AE related to DCV	(75)
nd GBM 34/34/0 42	Tumor lysate	IFNγ, LPS	i.n.	up to 15×: 4× weekly, 5× monthly, 3-monthly 1–5 × 10^6^	18.8 m/6.8 m		More AE in DCV group; more TMZ related AE in DCV group (thrombocytopenia more frequent in DCV); related to DCV: local pain, local reactions, fever, joint pain, general weakness	(76)
nd GBM 43/43/0 42	Tumor lysate	IFNγ, LPS	i.n.	Up to 15×: 4× weekly, 5× monthly, 3-monthly 1–5 × 10^6^		- Increase Increase		(77)
nd GBM 27/27/0 20	Tumor lysate of irradiated tumor cells	-		10×: 4× biweekly, 6× monthly 20–50 × 10^6^	31 m/–			(78)
nd GBM 232/232/0 99	Tumor lysate	–	i.d.	3× every 10 days, 3× monthly, every 6 months 2.5 × 10^6^	23.1 m/–		2.1% of pts grade 3/4 AE related cerebral edema, seizures, nausea, lymph gland infection; non-serious: injection site reactions, fatigue, low-grade fever, night chills	(79)
nd GBM 24/24/0	Tumor lysate	TNFα, IL-1β, IL-6, PGE2	i.d.	4× biweekly, 2× monthly, 1× 2 month later 5/10 × 10^6^	20.1 m/10.5 m	- Increase NK cell response	1 pulmonary embolism, 1 deep venous thrombosis + embolism, 1 disseminated intravascular coagulation, seizures, convulsion, myositis, skin reaction with itching, erythema, urticaria, inflammation	(80)
nd/rec GBM 22/13/9 21	CSC lysate	–	i.d.	3× weekly 2–4 × 10^6^	13.7 m/6.9 m^12^	- Increase -	Mild fever, erythema	(81)
rec GBM 20/0/20	Tumor lysate	TNFα, IL-1β, IL-6, PGE2	i.d.	3× biweekly, 2× monthly 20/10/5 × 10^6^		- Increase -	Brain edema, vomiting, asthenia, seizure, dysphasia, dizziness, cognitive disturbance, hyposthenia, vaccination site reaction: erythema, pruritus, pain, induration	(82)
nd GBM 81/81/0 43	Peptides^4^	IFNγ, LPS	i.d.	4× weekly, 4× monthly, every 6 months 11 × 10^6^	17 m/11.2 m	- 34/68 -	No AE related to DCV; fatigue, convulsions, nausea	(83)
nd GBM 23/23/0	CMVpp65 mRNA	TNFα, IL-1β, IL-6, PGE2	i.d.	3× biweekly, monthly 20 × 10^6^	41.1/41.4 m/–			(84)
nd HGG 16/14/0	Peptides^5^	TNFα, IL-1β, IFNα, IFNγ, poly(IC)	i.d.	3× weekly, 2× biweekly, 5× monthly 10–50 × 10^6^	19 m/11 m	27% 67% -	Only ≤grade 3	(85)
nd/rec GBM 5/3/2	Individual TAA mRNAs	TNFα, IL-1β, IL-6, PGE2	i.d.	3–8×: 2–4 weeks of intervals			No severe AE; others: skin rash, fever	(86)
rec GBM 1/0/1	CMVpp65		i.d.	3× weekly 5 × 10^6^			No grade 3/4; mild fever, lymphopenia (TMZ)	(87)

rec, recurrent; nd, newly diagnosed; HGG, high grade glioma (grade III and IV); EGFRvIII, epidermal growth factor receptor variant III; KLH, keyhole limpet hemocyanin; IL-13Rα2, interleukin-3 receptor α2; CSC, cancer stem cells; mRNA, messenger ribonucleic acid; CMV, cytomegalovirus; WT-1, Wilms’ tumor 1; PB, peripheral blood; TNFα, tumor necrosis factor α; IL-1β, interleukin-1β; PGE2, prostaglandin E2; OK432, preparation of streptococcus pyrogenes; IFNγ, interferon-γ; MCM, monocyte-conditioned medium; IL-6, interleukin-6; IFNα, interferon-α; poly(IC), polyinosinic:polycytidylic acid; LPS, lipopolysaccharide; i.d., intradermal; s.c., subcutaneous; i.t., intratumoral; i.n., intranodal; i.v., intravenous; SD, stable disease; PR, partial response; MR, mixed response; CR, complete remission; OS, overall survival; PFS, progression-free survival; DTH, delayed-type hypersensitivity.

^1^EphA2, IL13Rα2, YKL-40, GP100.

^2^WT-1, HER2, MAGE-A3, MAGE-A1, GP100, KLH.

^3^HER2, TRP-2, GP100, MAGE-1, IL-13α2, AIM-2.

^4^TRP-2, GP100, HER-2/NEU, Survivin.

^5^MAGE-1, AIM-2, HER2, TRP-2, GP100, IL13Ra2.

^6^WT-1, HER2, MAGE-A3, MAGE-A1, GP100, (KLH).

^7^In all studies, monocyte-derived DC were used.

^8^OS/PFS calculated from data provided in the manuscript.

^9^Toxicities have not clearly been attributed to DC vaccination.

^10^IFNγ responses have been detected by ELISA, enzyme-linked immuno spot (ELISPOT) assay, intracytoplasmic staining and flow cytometry, or quantitative PCR (qPCR).

^11^Others include proliferative or cytotoxic responses towards targets, tetramer staining, and flow cytometry and increase in GM-CSF, TNFα, IL-2, and IL-17a secretion upon specific restimulation and in tetramer-staining cells.

^12^Manuscript did not discriminate between GBM and grade III tumors or newly diagnosed and recurrent GBM.

Most patients underwent cytoreductive surgery prior to DCV, but patients who were biopsied only or had no surgery at all underwent treatment as well. An association of survival and the extent of resection, which by itself is predictive for better survival (89), has also been reported for DCV (78), and a state of minimal residual disease has been indicated to be beneficial for vaccination therapy (44, 48, 65, 78). This may be due to a reduction of local immunosuppression, which correlates with the tumor size (90, 91), and the sheer number of fast growing tumor cells, which otherwise would have to be eliminated by the CTL. However, in another study, the extent of resection was not associated with survival (76), so a more detailed comparison of the absolute residual tumor volume and, in particular, the composition of the tumor [e.g., the contribution of an immunosuppressive tumor microenvironment (TME)] is required.

Therapies concomitant to DCV included radio- and chemotherapy, mainly with TMZ (4); but in several trials, DCV has been used as the sole treatment. There may be advantages but also disadvantages for vaccination in the context of TMZ chemotherapy (see also the section *Dendritic Cell Dose, Vaccination Schedule, and Route of Application*).

In the previous trials, mainly patients with newly diagnosed or recurrent GBM have been treated with DCV, but in several studies, grade III tumors were also included (**Table 1**). It is unknown whether there is a difference in the responsiveness of grade III and grade IV tumors to DCV; however, a trend for a higher immunological response rate in newly diagnosed patients has been reported (50), which may be due to the less heavy pretreatment of those patients.

Overall, DCV was well tolerated (**Table 1**). Severe side effects (≥grade 3) attributable to vaccination have not been observed except for one patient with gross residual tumor post-surgery, who suffered from peritumoral edema, which was controllable by glucocorticoids (41, 48). Other severe side effects have been consistent with either the respective concomitant therapies or disease progression. Frequently observed mild and easily controllable toxicities (≤grade 2), which may be attributable to DCV, are injection site reactions with itching, pain, erythema, induration, and lymph node swelling as well as flu-like symptoms, fever, fatigue, myalgia, headache, edema, and meningeal irritation, which, however, can also be observed in the course of other concomitant therapies or be due to the disease. Thus, overall toxicity of DCV therapy is limited. However, it has to be noted that Mitchel et al., who vaccinated a GBM patient with DC transfected with cytomegalovirus (CMV) phosphoprotein 65 (pp65) mRNA and applied the vaccines intradermally together with GM-CSF, reported induction of a type I hypersensitivity-like reaction with IgE, but also IgM and IgG antibodies against the GM-CSF (92), which however resolved when vaccination was continued without GM-CSF. On the one hand, these results document the potency of DCV to induce immune responses. On the other hand, however, they urge caution in using any type of protein supplement at higher doses injected together with the DC.

Induction of antigenic target-directed immune responses have been observed in the course of DCV (**Table 1**), with detection of IFNγ responses being most informative (50, 71, 77, 82, 83, 85), but antitumoral cytotoxic responses (35, 42–44) and an increase in tetramer positive cytotoxic T cells (43, 56, 61, 67, 72–74) have been reported as well.

Several studies identified immunological responders based on antigenic-target directed delayed-type hypersensitivity (DTH) reactions, IFNγ responses, or cytotoxic responses, which increased in the course of vaccination and reported longer survival times for responders (44, 45, 50, 55, 66, 77, 81, 82). Indeed, in the study by Wheeler et al., immunological responders (IFNγ qPCR) had a significantly longer OS (599 *vs.* 401 days) and time to progression (260 *vs.* 146 days), and the 2-year OS rates compared favorably (56% *vs.* 8%) for the responders compared with the non-responders (50). Moreover, long-term survivors with durable IFNγ responses have been identified (71). However, although there are reports of an association of detectable antitumoral immune responses and better clinical outcome, this is not true for all studies (67, 75). Indeed, there appears to be no strong correlation between detection of systemic antitumoral immune responses and clinical outcome, which may be due either to the parameters tested or to a failure of the systemically detectable response in reaching the brain and effectively killing the tumor cells.

Survival of vaccinated patients compared favorably with matched or historic controls (35, 44, 45, 51, 54, 57, 68, 71, 85, 86). For newly diagnosed patients, mOS ranged from 15 to 41.4 months, and the progression-free survival (PFS) ranged from 6 to 25.3 months (**Table 1**).

Meanwhile, several controlled studies have been published, six of them randomized (60, 62, 76, 79, 81, 83), whereas Batich et al. summarized the data of three previous similar trials they conducted (84) (for details, see **Table 1**).

For a phase III trial on 331 newly diagnosed GBM patients (232 in the DCV group and 99 in the control group), Liau et al. described long-term survivors and a mOS of all patients of 23.1 months. Unfortunately, however, they did not yet report conclusive data on the outcome of the study.

In two randomized phase II trials with 34 (60) and 25 (62) newly diagnosed GBM patients, mOS (31.9 and 17 months) of the vaccination groups was significantly improved compared with that of the respective control groups (15 and 10.5 months).

In another randomized phase II trial with 41 newly diagnosed and recurrent GBM patients (81), Yao et al. reported that DCV significantly prolonged mOS (13.7 *vs.* 10.7 months).

In two randomized phase II trials with 76 (76) and 124 newly diagnosed GBM patients (83), no significant differences in mOS (18.8 *vs.* 18.9 months and 17 vs. 15 months, respectively) between patients in the DCV and control groups were observed, although Wen et al. reported a significantly improved PFS for the vaccinated patients (11.2 *vs.* 9 months).

Batich et al. (84) merged their data on DCV with CMVpp65 mRNA transfected DC of newly diagnosed GBM patients (71, 74), either admixed with GM-CSF (11 patients) or tetanus-diphtheria toxoid (Td; six patients) conditioning of the vaccination site. They reported a mOS of vaccinated patients of 41.1 (GM-CSF) and 41.4 months (Td) compared with 18.5 months for control patients receiving unpulsed DC (six patients). Moreover, they describe long-term survival rates at 5 years of 36.4% and 33.3%.

Thus, even from these controlled trials, which revealed mixed results, it is still difficult to draw a conclusion as to the efficacy of DCV in GBM. Since markers of immunosuppression such as the PD-1^+^:CD8^+^ ratio (78), the presence of regulatory T cells (Treg) or CTLA-4 expression pre- and post-vaccination (67, 93), and an immunocapability score (77) are associated with survival after DCV, combining DCV with tools interfering with immunosuppression seems to be called for to improve efficacy. Moreover, to improve DCV efficacy may also require further optimization of the DC vaccine in respect to target antigen selection, preparation of the cells, the integration of DCV into other treatment regimens, and dosing and scheduling of the vaccination(s).

## Antigenic Target

Efficacy of DCV depends on the presence of TAA or so-called neoantigens in the individual tumor, which allow specific recognition and killing of the tumor cells. The overall mutational load—the frequency of neoantigens—of this tumor entity is low, and the majority of GBM (>85%) contain only up to 10 mutations/1.4 Mb, except for patients with recurrent tumor after TMZ chemotherapy, which increases the mutational load [(94); for review, see (95)]. Nevertheless, multiple TAA have been identified for GBM (96–99).

In previous DCV studies, tumor lysates, apoptotic bodies of tumor cells, irradiated tumor cells, tumor mRNA, and fusions of tumor cells and DC as well as peptides eluted from the surface of tumor cells have been used as whole-tumor cell sources of TAA (**Table 1**). They are produced from the patient’s own tumor obtained from surgery and can also be derived from subsets such as cancer stem cells (68, 81), which could provide a therapeutic advantage (28, 29, 100). Whole-tumor cell sources of TAA most likely will contain multiple TAA, which are present in the individual tumor of a patient (including the various cellular subsets within the tumor), i.e., the patient’s full antigenic repertoire, ensuring antigenic diversity, thereby reducing the risk of escape of TAA-loss variants (101). Since the respective proteins are endogenously processed in the DC, in contrast to e.g., synthetic HLA class I restricted peptides, presentation on HLA class I and II molecules is possible and independent of the HLA type of the patient, thereby allowing induction of CTL as well as T_H_ responses at the same time, which is a prerequisite for the development of an efficient CTL response. At least for tumor lysates, presentation/cross-presentation *via* HLA class I and II molecules of lysate-derived peptides has been shown (102). The observation of CTL responses after DCV is further evidence that the material taken up by the DC is indeed cross-presented *via* HLA class I (**Table 1**). Furthermore, whole-tumor cell sources of TAA, which have not been processed or cultured extensively, may provide the so far unknown necessary signals, allowing the DC to guide effector T cells to the brain (103).

TAA represent only a small fraction of proteins; therefore, a low tumor content of the tumor sample used for preparation of the TAA would even further reduce the TAA concentration. While this may not pose a higher risk to the patients, it may compromise efficacy of the vaccine. Therefore, a high tumor cell content of the sample has to be ensured, a task that will benefit from fluorescence-guided surgery, which allows intraoperative identification of the “solid” part of the tumor (63). Nevertheless, an estimate of the tumor cell content should always be obtained and reported when publishing DCV data, to establish values relevant for efficacy, which are currently unknown. When using protein solutions, such as lysates, the protein concentration used to load the DC should also be determined and reported. Despite the abundant presence of normal self-antigens in whole-tumor cell sources of TAA, induction of autoimmunity or other severe side effects attributable to its use for vaccine production have not been reported (**Table 1**). However, when using whole-tumor cell sources of TAA, it has to be kept in mind that they may inhibit vaccine production, i.e., DC differentiation and maturation, or modify the function of the resulting DC, because of the presence of immunosuppressive factors produced by the tumor cells (104). Moreover, efficacy appears not to be based alone on the source of TAA, but also on how it is processed. Gark et al. reported that induction of immunogenic cell death prior to DC-loading increases survival of animals substantially and shifts responses in the brain towards T_H_1/CTL/T_H_17 (105).

As an alternative to whole-tumor cell sources of TAA, molecularly defined TAA such as specific peptides, proteins, and DC transfected with the respective target antigen mRNA have been used for DCV of GBM (**Table 1**). Molecularly defined TAA represent a more standardized, consistent, and reproducible source of TAA and offer the advantage of higher available target antigen concentrations and lower background, and target-specific responses can be easily monitored. They can even be produced personalized (86). Nevertheless, multiple molecularly defined TAA should be used to reduce the risk of TAA-loss variants escaping immune control (101).

It is currently unknown whether whole-tumor cell sources of TAA or molecularly defined TAA (and which ones) are superior in inducing antitumoral immune responses and more beneficial clinically in GBM. Irrespective of the source of TAA, induction of antitumoral T-cell responses by DCV has been reported in previous studies (**Table 1**). The controlled studies, which documented a clinical benefit, used tumor lysates, thus a whole-tumor cell source of TAA (60, 62, 81), as well as CMVpp65 transfected DC, thus a molecularly defined TAA (84). Similarly, in the controlled studies that did not report a clinical benefit, either tumor lysates (76) or a set of six defined peptides (83) was used.

When peptide and tumor lysate-loaded DC were compared in animal models, superior efficacy has been reported for lysate-loaded DC (106). Moreover, Neller et al. concluded from the analysis of 173 published immunotherapy trials on various tumor entities, including melanoma, renal cell and hepatocellular carcinomas, and lung, prostate, breast, colorectal, cervical, pancreatic, and ovarian cancers a higher objective response rate (8.1% *vs.* 3.6%) when whole-tumor cell TAA were used compared with molecularly defined TAA (107). Thus, there appears to be an advantage of whole-tumor cell sources of TAA. However, particularly results from DCV against CMVpp65, a target that may be present in the majority of GBM patients and appears not to be expressed in normal brain cells (108, 109), generated convincing results for the efficacy of a molecularly defined TAA (71, 74, 84).

## Dendritic Cell Vaccine

In 1994, Sallusto et al. reported the generation of immature DC with high antigen uptake activity from blood monocytes using the cytokines GM-CSF and IL-4, which then could be matured by an additional culture period with TNFα, resulting in cells with potent T-cell stimulatory activity (110). These so-called monocyte-derived DC are potent stimulators of naïve CD4^+^ T_H_ cells and can polarize T_H_-cell responses towards T_H_1, cross-present antigens, and activate CD8^+^ CTL [for review, see (14)]. Yet their activity depends on the maturation or activating stimulus.

Although there are now techniques to enrich the various rare blood DC populations, which may also be promising candidates for DCV (111), monocyte-derived DC have been used in all DCV trials in GBM to date (**Table 1**). Monocytes were enriched from either peripheral blood or leukapheresis products by adherence, immunomagnetic selection of CD14^+^ cells, immunomagnetic depletion of B-cells and T cells, or elutriation. Depending on the enrichment procedure, monocyte purity varied, with CD14^+^ selection yielding the highest purity. Higher monocyte purity may result in more stable culture conditions, reduces modulating effects of contaminating cells, and yields higher-purity DC preparations. Overall, production of the vaccine is more reproducible and results in a more homogenous cell population.

Typically, monocytes were cultured with GM-CSF and IL-4 in a first culture phase, generating immature DC within ~6 days. The antigen-uptake activity of immature DC is well developed. Therefore, they have been used for antigen loading, when tumor lysates, apoptotic bodies of tumor cells, irradiated tumor cells, fusions of tumor cells and DC, proteins, and mRNA transfection were used as source of TAA, whereas peptide pulsing was performed mainly with mature DC.

Immature DC are poor stimulators of T cells and can even induce tolerance. Only when they are activated, e.g., by proinflammatory signals, do they develop into mature DC (14, 111). Originally, TNFα has been used as maturation factor (110). In 1997, Jonuleit et al. described a more potent maturation stimulus, a cytokine cocktail containing IL-1β, IL-6, and TNFα together with PGE2 (112). This cocktail and other combinations of these factors with or without factors such as type I and II interferons, lipopolysaccharide, and toll-like receptor ligands [e.g., polyinosinic:polycytidylic acid (poly(IC))] have been used in the clinical studies summarized here (for details, see **Table 1**).

The optimal maturation stimulus is still a matter of debate and since immature DC sense, integrate, and translate environmental changes into signals to the T cells, differences in medium, cell density, the frequency of dead cells in cultures, etc., may result in different outcomes in regard to target cell function. Moreover, the same factors can have beneficial as well as adverse effects. For example, the combination of lipopolysaccharide and IFNγ induces semi-mature DC, which produce IL-12 and induce CTL responses (113), but it also appears to initiate an immunosuppressive program with the induction of indoleamine-2,3-dioxygenase [IDO; (114)]. Similarly, PGE2, which is part of a potent cytokine cocktail inducing DC maturation (112) and improves the migratory response of DC (115), can also induce IDO (116). In addition, the effects of the maturation factors can be further modulated by the source and processing of the TAA (104, 105). Thus, outcome of DC maturation is difficult to generalize. Therefore, the functional properties of the cells have to be determined for the respective conditions used in each manufacturing process.

Mature DC can be distinguished from immature DC by their expression of the surface molecules CD83 (117) and CD25 (118), whereas other markers such as CD40, CD80, CD86, and HLA-DR may differ only in expression density (118). Indeed, high-density expression of CD80 appears to be of utmost importance because it interacts in-cis with PD-L1, thereby blocking PD-L1 binding to PD-1 on T cells and inhibition of T-cell activation (119). However, identification of “mature” DC may be even more complex. Previously, a population of mregDC (mature DC enriched in immunoregulatory molecules) has been identified, which co-expresses maturation markers including CD83, CD40, CD80, CD86, and RelB together with immunoregulatory genes such as PD-L1, Pdcd1lg2, CD200, Fas, Socs1, Socs2, and Aldh1a2 (120).

Overall, mature DC for immunotherapy should 1) have potent T_H_-cell and CTL stimulatory activity; 2) polarize responses towards T_H_1, which is required for efficient induction of effector CTL (121, 122); 3) have to imprint effector T cells for brain tumor homing, which may require induction of VLA-4 (α4/β1 integrin), which appears to be the main integrin for lymphocyte trafficking to the brain (123); 4) express CCR7, which is required for lymph node homing (124, 125); 5) be phenotypically stable upon withdrawal of cytokines (126) to prevent re-differentiation towards immature (and possibly tolerogenic) DC after administration; 6) be resistant to immunosuppressive cytokines like TGF-β (127); and 7) not induce tolerance. Particularly, the induction of target antigen-specific tolerance, which has been reported for immature and semi-mature DC, but not fully mature DC would be detrimental to the intended induction of antitumoral immune responses (128). Indeed, de Vries and colleagues showed that TAA-loaded mature DC, but not immature DC, induce immunological responses in melanoma patients (129), and even more important, Dhodapkar et al. documented a decline of the influenza matrix peptide-specific T-cell response in healthy individuals after vaccination with matrix peptide-loaded immature DC (130).

In GBM, several clinical trials used immature DC as vaccines (**Table 1**). Somewhat unexpectedly, immunological responses as well as beneficial effects on survival have been reported, although Yamanaka et al., who used mature as well as immature DC in their study, reported a trend towards a better outcome in GBM patients vaccinated with the mature DC (45). Two of four controlled trials reporting a clinical benefit used immature DC (60, 81), whereas in the remaining two trials, DC were matured with TNFα, IL-1β, and PGE2 (62) or TNFα, IL-1β, IL-6, and PGE2 (84). Interestingly, both controlled trials that did not document clinical efficacy (76, 83) used lipopolysaccharide + IFNγ for maturation, thus a factor combination that also results in the induction of IDO (114). In conclusion, currently, the most potent vaccine for DCV of GBM has not been identified, and even the use of immature DC cannot be excluded, although there are strong arguments for the use of mature DC.

## Dendritic Cell Dose, Vaccination Schedule, and Route of Application

The minimum DC dose reported to elicit T-cell responses in healthy individuals is 2 × 10^6^ DC/vaccine (16). A wide range of DC doses have been used in GBM-DCV trials (0.25–100 × 10^6^ DC/individual vaccine, **Table 1**). In four controlled studies, which reported a significant survival benefit for vaccinated patients, doses of 2–4 × 10^6^ (81), 6 × 10^6^ (62), 20 × 10^6^ (84), and 20–50 × 10^6^ (60) DC/vaccine were used, while doses of 1–5 × 10^6^ (76) and 11 × 10^6^ DC/vaccine (83), i.e., in a comparable range, resulted in no clinical benefit in two other controlled trials. In several studies, immunological responders were identified based on an increase in IFNγ (qPCR, ELISPOT) after DCV, and immunological responsiveness was positively associated with survival. DC doses ranged from 1 to 50 × 10^6^ DC (50, 71, 77, 85). No correlation with DC dose has been described for either clinical outcome or immunological responsiveness, and a dose–response relationship with an optimal dose cannot yet be defined. Because dose-limiting toxicity has not been reached in previous studies, doses tend to be maximized based on the number of cells available from the production process and the vaccination scheme. In a dose-escalating study by Prins et al. using 1, 5, and 10 × 10^6^ DC/vaccine, no association was found between increasing DC dose and toxicity or immunologic response, but longer survival (though not statistically significant) was observed in those patients receiving the lowest DC dose (57). Although only a fraction of the injected DC reaches the lymph nodes (131, 132), this may still be far more than, e.g., in the case of infections and could actually be too much. However, Mitchel et al., who used fairly high DC doses/vaccine (20 × 10^6^), reported an improved survival of patients, when the efficiency of migration of DC to the lymph nodes was enhanced (71). Moreover, intranodal application, which may allow to directly deliver even higher DC numbers to the lymph nodes, resulted in increased IFNγ responses after vaccination (55, 56, 77), although Buchroithner et al. could not document a survival benefit in a controlled trial using this approach (76). However, it has to be kept in mind that DC vaccines are likely to differ in potency, e.g., immature as well as mature DC have been used, and the vaccination schedule and route of application, as well as many other parameters, also influence efficacy, so an optimal DC dose is difficult to define and may need to be determined on a case-by-case basis for each vaccination strategy, yet it has the potential to improve efficacy.

Multiple vaccinations were given per patient, mainly 3–10, but also up to 23 (**Table 1**). Thus, many studies used a prime and boost vaccination approach. Currently, it is not clear whether multiple vaccinations improve the outcome. Jouanneau et al. showed in a GL26 orthotopic tumor model that multiple injections of TAA-loaded DC did not further improve the outcome, whereas a lysate boost resulted in a significantly prolonged survival and was associated with an increased CTL response and antibody formation, contributing to the therapeutic effect (133). Indeed, de Vleeschouwer et al. described a trend of prolonged PFS with a DCV strategy with lysate boosting, although the contribution of the lysate boosts remains unknown (48). In contrast, Okada et al. reported that DC boost vaccination further enhanced IFNγ responses (56) and Buchroithner et al. described a trend towards better survival in patients receiving more vaccines (76). Thus, the number of vaccinations and the use of DC vaccines or lysates (or any other target antigen such as e.g., peptides) for boost vaccination are parameters that influence efficacy and remain to be optimized.

Vaccines have been administered weekly, biweekly, or monthly, or in combinations thereof, frequently integrated into established treatment regimens of radiotherapy and chemotherapy. The standard of care for newly diagnosed GBM consisting of resection, and radiotherapy with concomitant and subsequent adjuvant TMZ chemotherapy (4) appears to offer several windows of opportunity for vaccination, allowing to retain the standard of care with proven efficacy while integrating DCV and potentially exploiting synergies between the two therapeutic approaches.

Immunosuppression, which is prominent in GBM (see below), correlates with tumor size, and surgical cytoreduction can at least partially restore immunological responsiveness (90, 91). Fluorescence-guided surgery (FGS) allows to increase the extent of resection safely, and radiologically complete resections could be performed in 65% of patients compared with 35% in a control group undergoing standard surgery (89) that was associated with improved survival. Thus, integrating FGS into a DCV approach (63, 75) may not only minimize residual tumor mass and thereby also immunosuppression, both of which are beneficial for immunotherapy (44, 48), but because of the extended PFS, it may also prolong the time period available for T-cell responses to clear residual tumor cells before the tumor mass becomes too large again. Moreover, FGS ensures high tumor cell frequency in the tumor samples, because the vital “solid” part of the tumor can be identified intraoperatively (63), an advantage when whole tumor cell sources of TAA such as tumor lysates are used. The substantial increase in the extent of resection by FGS may also improve safety of DCV, because in a patient with gross residual tumor after standard surgery, a grade 4 peritumoral edema has been reported, which was considered to be associated with DCV (41, 48). In addition, a maximal resection generally allows to wean glucocorticoids faster, which are perioperatively applied in GBM patients to reduce swelling in the brain—a possible advantage for DCV due to the immunosuppressive activities of the steroids. However, although glucocorticoids are potent inhibitors of T-cell immunity (134) and Keskin et al. reported induction of polyfunctional T cells after peptide vaccination only in those GBM patients that did not receive dexamethasone (135), its role during DCV is not entirely clear. Therefore, it should be used judiciously or excluded, until more data on dosing and timing of glucocorticoid use during DCV of GBM patients become available (136). Indeed, in four of the controlled trials (79, 81, 83, 84), the use of glucocorticoids was either excluded entirely or only minimal doses of 2–4 mg/day of dexamethasone were allowed.

Vaccination is either performed in the time period between radiochemotherapy and adjuvant TMZ or in the course of the adjuvant TMZ cycles around day 21 (137), because there appears to be a rationale to combine DCV with TMZ chemotherapy: 1) TMZ can improve immunological responsiveness (138–140), probably by reducing Treg (see below) (139, 141) and interfering with their recruitment to the tumor (142). 2) Although it frequently causes lymphopenia, the recovering lymphocyte compartment after chemotherapy has been shown to allow for efficient induction of antitumoral responses (143–145). 3) Dying tumor cells after radiochemotherapy or chemotherapy lead to a release of tumor antigens, which could enhance homing of tumor-specific effector CTL to the brain tumor after luminal presentation of the target peptides on HLA class I molecules on the cerebral endothelium (146). However, effects appear to depend on TMZ dose; e.g., lower but not higher TMZ doses were shown to deplete Treg (141), whereas myeloablative but not non-myeloablative doses enhanced responses to a peptide vaccine (147). In addition, results from Pellegatta et al. indicate that adjuvant TMZ may deplete CD8^+^ T cells previously expanded by DCV, because in contrast to NK cells, they fail to express the multidrug resistance transporter protein ABCC3 (80). A decline in responding cells after adjuvant TMZ has also been described by Batich et al. (84). Moreover, is has been shown that DCV only in the absence of TMZ, although with additional conditioning of the injection site with tetanus toxoid, results in the generation of T effector memory cells producing IFNγ, which is positively associated with survival (82). These results would argue against combining DCV and TMZ chemotherapy. Because all controlled DCV trials in GBM used TMZ in both arms, currently, it cannot be determined whether or not it affects efficacy (60, 62, 76, 81, 83, 84).

Effective induction of antitumoral T-cell immunity requires the DC to reach the T-cell areas of lymph nodes. Although the cervical nodes (148–151) or the nasopharynx-associated lymphoid tissue (152) serve as lymph node stations of brain immune responses, imprinting the brain homing phenotype of effector T cells is a function of the DC rather than that of a distinct lymph node. Therefore, effective responses targeting antigens in the brain can also be initiated in other lymph nodes besides the cervical nodes or the nasopharynx-associated lymphoid tissue (103).

Depending on the route of application, DC can be detected in different organs. Intravenous application results in a rapid enrichment in the liver, lungs, and kidneys but is the highest in the spleen, whereas after subcutaneous application, there is a marked accumulation of DC in the draining lymph nodes, with a preferential paracortical localization in the T-cell areas (153). When intradermal application is used instead, even more DC reach the T-cell areas of the lymph nodes in mice (154) as well as in humans (155), with only mature but not immature DC efficiently migrating to the lymph nodes (156). This is probably due to the expression of the CCR7 chemokine receptor on the mature DC (118, 124, 125) and the responsiveness of the cells to the chemokines CCL19 and CCL21, which are expressed constitutively by peripheral lymphatic endothelial cells and lymph node stromal cells (157).

DC can already be detected in the lymph nodes 30 min after injection, there is a maximum after 48 h, and they appear to persist for up to 14 days (158–160). Only ~5% of injected DC may reach the lymph nodes, which appears to be sufficient for effective induction of antitumoral immune responses (131, 132), although substantially higher values have been reported as well (160). A large fraction of DC remains at the injection site, rapidly becomes apoptotic, and is cleared by CD163^+^ macrophages (131). However, it is possible to augment DC migration to the lymph nodes by preconditioning the application site with a potent recall antigen such as tetanus/diphtheria toxoid, associated with improved survival of patients (71).

The life span of DC in the lymph nodes is limited to a few days (161, 162), and they may be removed by apoptosis (163, 164) and phagocytic clearance by macrophages (165). However, endogenous DC in skin and lymph nodes may prolong antigen presentation beyond the life span of the injected DC (166).

Overall, there appears to be an advantage of intradermal application of vaccines, and indeed, most studies have used it (**Table 1**). Whether the higher DC numbers delivered directly to the lymph nodes by intranodal application (55, 56, 76) are even more effective remains to be determined.

There is also the possibility of intratumoral application of DC. Pellegatta et al. have documented in an orthotopic GL261 glioma model that the efficacy of intratumoral application of GL261 lysate-loaded DC is lower than that of subcutaneous application, but that the combination of both procedures significantly improves survival (167). Since intratumorally administered DC remain in the brain parenchyma and were not detected in the cervical lymph nodes, a different mechanism than for the subcutaneously administered DC appears to be responsible for improved survival. Whether they contribute to the final maturation and shaping of the effector T-cell response (168) by acting as tissue inflammatory DC or reduce tumor cell growth in this model because of their production of TNFα (167) remains to be determined. Intratumoral application has not yet been studied in clinical trials in GBM.

## Immunosuppression and Immune Checkpoint Regulation

**Figure 1:**
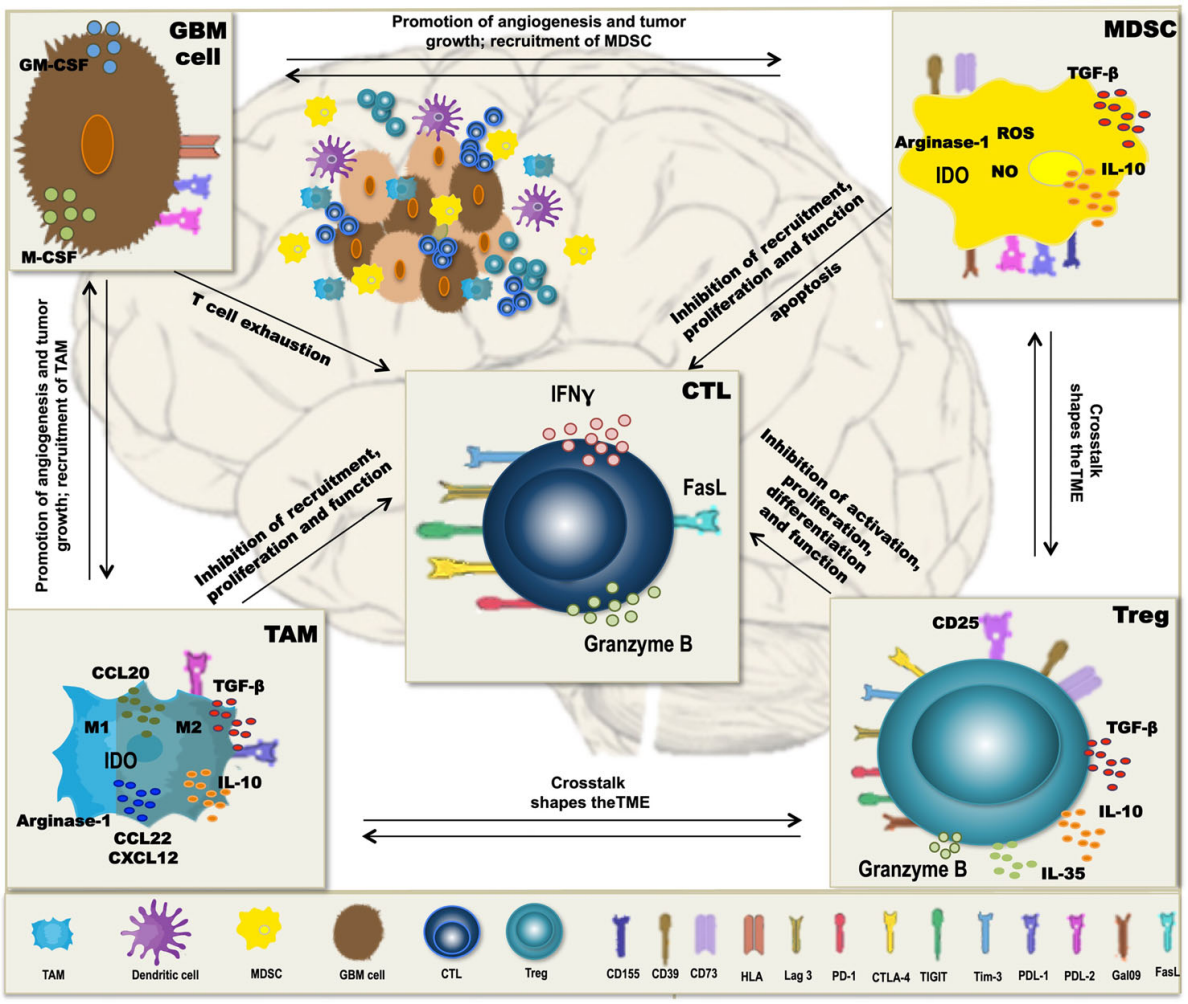
Mechanisms of immunosuppression in glioblastoma (GBM). In GBM, tumor-associated macrophages (TAM), myeloid-derived suppressor cells (MDSC), and regulatory T cells (Treg) form a potent immunosuppressive tumor microenvironment (TME), which inhibits antitumor immunity and thereby interferes with dendritic cell vaccination (DCV). Besides the intrinsic immune escape mechanisms of the tumor cells, immune checkpoint molecules, like PD-L1, PD-L2, Tim-3, Lag-3, CD155, and galectin-9 that normally control the extent of immune responses, are expressed on the immunosuppressive cells of the TME, contributing to T-cell dysfunction and subsequently inefficient antitumoral immune responses. Cells of the TME secrete cytokines such as TGF-β, IL-10, and IL-35 and the chemokines CCL20, CCL22, and CXCL12, which inhibit T-cell proliferation and function and contribute to a crosstalk between the different TME cell types, thereby further enhancing immunosuppression. Additional mechanisms include the activity of indoleamine-2,3-dioxigenase (IDO) and arginase-1 as well as production of reactive oxygen species (ROS) and nitric oxide (NO), all interfering with a proper differentiation, expansion, and function of effector T cells.

**Figure 2:**
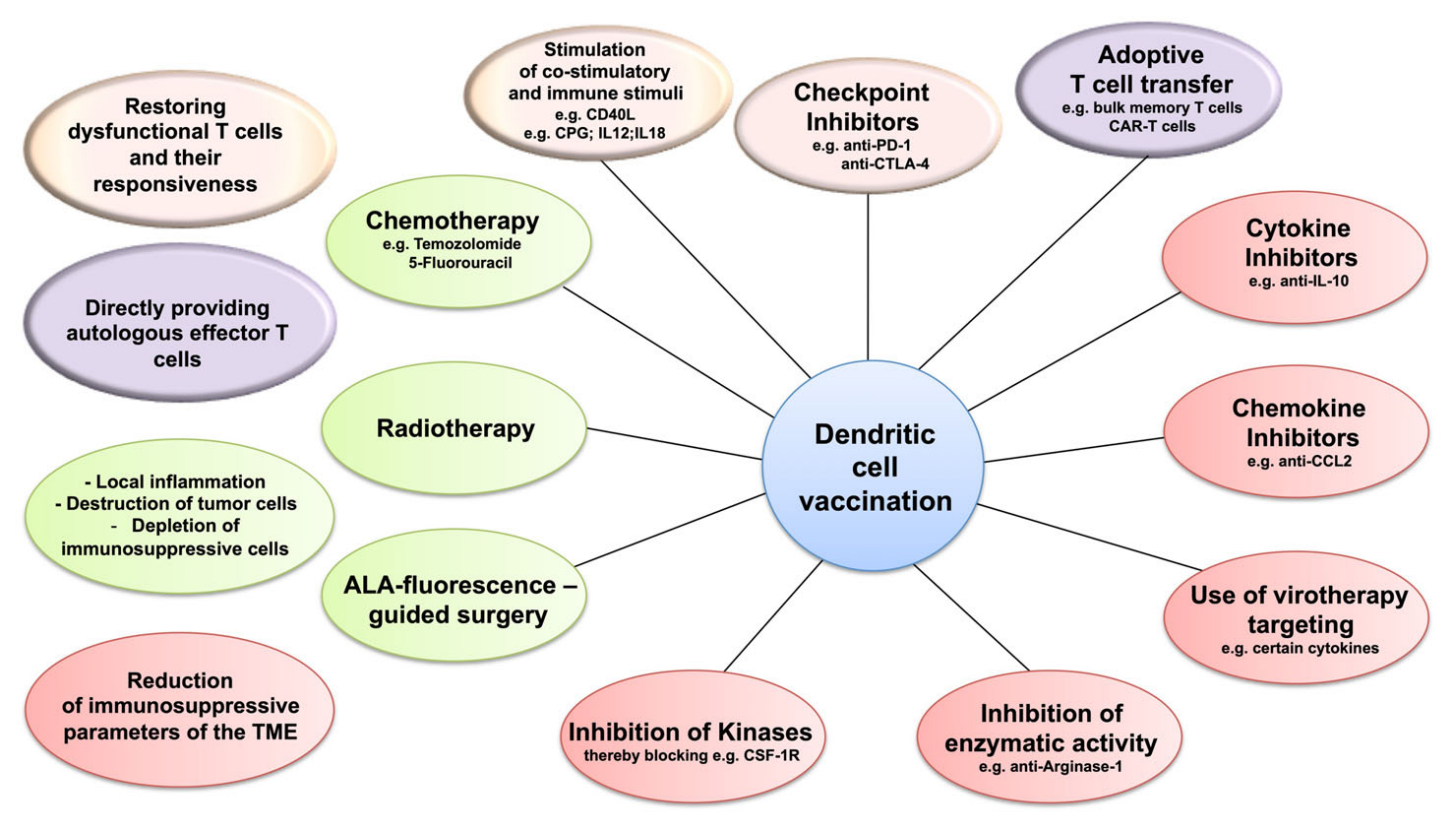
Dendritic cell vaccination (DCV) and targeting the immunosuppression in the tumor microenvironment (TME). Combining DCV with therapies targeting the three immunosuppressive cell populations of the TME—regulatory T cells (Treg), tumor-associated macrophages (TAM), or myeloid-derived suppressor cells (MDSC)—might improve efficacy. Potential target strategies include restoring the responsiveness of the dysfunctional T cells (pink), applying effector T cells by adoptive transfer (purple), depleting immunosuppressive cells, and modulating the inflammatory conditions in the TME (green) and blocking the mechanisms of immunosuppression (red).

A major obstacle to the therapeutic vaccination of GBM with DC is that the antitumoral immune response must be elicited in the context of immunosuppression. Humoral and cell contact-dependent mechanisms originating not only from the tumor cells themselves (including intrinsic mechanisms of immune evasion) (169) but also from the cells of the TME, such as Treg, tumor-associated macrophages (TAM), and myeloid-derived suppressor cells (MDSC), can inhibit antitumor immunity (**Figure 1**). Moreover, immune checkpoints—control mechanisms that limit and thereby prevent excessive immune responses—may be activated in the TME, also resulting in inefficient responses and T-cell dysfunction. Whether DCV by itself can tip the balance towards immunity is unclear, but efficacy may require the combination with additional therapeutic strategies (**Figure 2**) to overcome the adverse effects of immunosuppression and immune checkpoint regulation.

### Regulatory T Cells

Treg are a distinct immunosuppressive T-cell subpopulation, which contribute to maintaining immunological tolerance, limiting excessive immune responses, and promoting homeostasis and tissue regeneration. They either develop in the thymus (thymic (t)Treg) as a distinct T-cell lineage or differentiate in the periphery (peripheral (p)Treg) from naïve T cells [reviewed in (170, 171)]. Naïve/resting Treg reside mainly in the blood and secondary lymphoid organs. They can be identified as CD3^+^/CD4^+^/CD25^low^/CD127^−/low^ T cells, which express the transcription factor FoxP3 (172–176), although FoxP3 may also be transiently expressed at low levels in the course of activation of human conventional CD4^+^ and CD8^+^ T cells (177). They further express CD45RA, CD62L, and CCR7 (CD197), whereas expression of CTLA-4 (CD152) and CD45R0 is absent on the naïve/resting Treg. Upon T-cell receptor stimulation, they differentiate into highly proliferative and suppressive effector Treg, which are characterized by a CD3^+^/CD4^+^/CD25^high^/CD127^−/low^/FoxP3^high^/CD45RA^−^/CD45R0^+^/CCR7^−^/CD62L^−^/CTLA-4^+^ immunophenotype [reviewed in (170, 178)], with delineation of cells further improved by the presence of CD15s on effector Treg, but not conventional effector T cells (179). Depending on the stimulatory context and the affinity of T-cell receptor recognition, there is induction of chemokine receptors (e.g., CCR2, CCR4, and CXCR3) and adhesion molecules (e.g., lymphocyte function-associated antigen-1 (LFA-1), integrin-α4, and integrin-β1), which guide them to their target sites, as well as of effector molecules, which mediate selective mechanisms of immunosuppression. Indeed, effector Treg can upregulate transcription factors associated with distinct T_H_-effector phenotypes, allowing suppression to be tailored to the respective polarized responses, and it may be further modulated by the local microenvironment at the target site (170, 178, 180–182).

Multiple mechanisms contribute to effector Treg function:

1. They secrete the immunosuppressive cytokines IL-10, TGF-β, and IL-35, which inhibit T-cell activation and proliferation either directly or by suppressing the stimulatory activity of DC, and contribute to the formation of tolerogenic DC and the generation of additional Treg (183–186). These effects are further enhanced by cytokine-mediated crosstalk between the immunosuppressive cells of the TME, e.g., TGF-β stimulates MDSC proliferation and suppressive activity (187). Moreover, TGF-β downregulates intercellular adhesion molecule 1 (ICAM-1) and vascular cell adhesion molecule 1 (VCAM-1) on blood vessels and thus inhibits conventional T-cell infiltration into the TME (188).
2. Treg release granzyme and perforin, which induce apoptosis of effector T cells (189, 190). Moreover, CD4^+^ T_H_-effector cells expressing death receptor 5 (DR5) can also be killed by Treg expressing the corresponding ligand TNF-related apoptosis-inducing ligand (TRAIL) (191).
3. Due to the high-density expression of high-affinity IL-2 receptors (CD25/CD122/CD132) on Treg, they act as an IL-2 sink and deprive conventional T cells of IL-2, which suppresses their expansion and differentiation to effector cells and may cause effector T cells to become anergic or apoptotic (192).
4. Treg can release adenosine nucleosides. They express CD39 (ectonucleoside triphosphate diphosphohydrolase-1) and CD73 (ecto-5′-nucleotidase), which together convert adenosine triphosphate to adenosine, with extracellular adenosine inhibiting DC antigen presentation as well as proliferation and cytokine secretion of activated T cells through the A2A receptor (193–195). It further promotes differentiation and proliferation of Treg, expansion of MDSC, and polarization of M2 macrophages [reviewed in (196)]. Treg also contain high levels of cyclic adenosine monophosphate, which they can transfer into conventional T cells *via* gap junctions, thereby inhibiting their proliferation and IL-2 production upon activation (197).
5. Effector Treg express several surface molecules, which interfere with the activation, proliferation, differentiation, and effector function of conventional T cells. CTLA-4 is an immune checkpoint regulator. It binds to CD80/CD86 on DC with higher affinity than CD28 on conventional T cells, leading to anergy, apoptosis, or even the conversion of the activated conventional T cells into Treg, due to the absence of the co-stimulatory signal. However, CTLA-4 not only competes with CD28 for CD80/CD86 binding but also depletes them from the surface of DC by transendocytosis, thereby further preventing co-stimulation (198, 199). Moreover, it induces IDO in DC and causes their conversion to tolerogenic DC. IDO is an enzyme that degrades the essential amino acid tryptophan to kynurenine. The resulting local tryptophan depletion as well as the interaction of kynurenine with the aryl hydrocarbon receptor prevents T-cell proliferation and can induce differentiation of CD4^+^ T cells into Treg (200–202). Other immune checkpoint regulators including PD-1 and its ligand PD-L1, lymphocyte-activation gene 3 (Lag-3), T-cell immunoglobulin mucin-3 (Tim-3), and T-cell immunoreceptor with Ig and ITIM domains (TIGIT) can also be expressed on Treg and contribute to immunosuppression (203–208) [reviewed in (209, 210)].

In various solid tumors, there is a high frequency of tumor-infiltrating effector Treg and particularly a high Treg : CD8^+^ T-cell ratio, which negatively correlate with prognosis (211), indicating that there is a naturally occurring antitumoral immune response. Treg cannot be detected in normal brain, and they are rare in low-grade brain tumors, yet despite lymphopenia, Treg frequencies are increased in the TME as well as in the blood of GBM patients (212–215). Frequencies may vary by GBM subtype, with the IDH^wildtype^ (Iso-citrate dehydrogenase) and mesenchymal subtypes having higher Treg frequencies than IDH^mutated^ (216) and proneural and classical GBM subtypes, respectively (217). It has been reported that there is an age-dependent increase in the Treg : CD8^+^ ratio, with a maximal increase in the 60–69 years age group, which coincides with the median age (64 years) of GBM patients at diagnosis (218). Nevertheless, the association of Treg frequency with prognosis is moderate at the most (188, 214, 215, 219), in contrast to CD4^+^ and CD8^+^ effector T-cell infiltrates, which are positively associated with survival (188). However, association appears to require the concomitant presence of a low immunosuppressive signature (220). Indeed, a negative association of the Treg : CD8^+^ T cell ratio with survival has been reported (221), which could indicate that there are patients with natural antitumoral immunity and that only in these patients are Treg associated with poor prognosis.

In mouse models of GBM, a time-dependent accumulation of Treg after implantation of tumor cells has been described (222–224). Interestingly, Treg numbers increased first in blood and later, but still in the asymptomatic phase, in the tumor tissue (223, 224). Thus, Treg appear to be recruited to the tumor already in an early phase, when tumor cell numbers are still low and are not a consequence of the immune system simply being overwhelmed by the tumor mass at a later phase of tumor development. The chemokines CCL2 and CCL22, which are produced by brain cells as well as GBM cells and cells of the TME, appear to be responsible for the recruitment of the CCR4^+^ Treg to the TME of GBM (142, 215, 225).

Treg-mediated immunosuppression has been targeted to enhance the efficacy of natural or induced antitumoral immunity mainly in preclinical models of GBM. This includes depletion of Treg by anti-CD25 antibodies (145, 222, 226, 227), interference with their immunosuppressive activity, e.g., by blocking surface molecules such as CTLA-4 (222, 228, 229), TIGIT (230), Tim-3 (231), and PD-1 (229, 230, 232) or enzymes like ecto-5′-nucleotidase (CD73) (233) and IDO (229), or by preventing the accumulation of Treg in the TME by blocking CCR4-mediated migration (225). Irrespective of the approach, these studies could document a beneficial effect, with increased survival and reduction of tumor burden associated with restoration of antitumoral immunity, particularly when different strategies were combined.

However, specific inhibition of Treg or of their functional activity may not be required. Higher doses of non-fractionated radiotherapy (234) and chemotherapy with low-dose TMZ (141) or cyclophosphamide (235) have also been reported to reduce Treg, although it is unclear whether the long-lasting lymphopenia induced by the standard concomitant radiochemotherapy of GBM patients is beneficial for antitumoral immunity (144, 145, 236). Moreover, immunogenic stimuli such as CPG oligonucleotides (237, 238), virotherapy (239, 240), or the use of agonistic antibodies specific for co-stimulatory receptors such as CD40 (241) and OX40 [CD134; (242)] may be sufficient to tip the balance towards antitumoral immunity, associated with a reduction in Treg. Another promising approach is to modulate the metabolism of Treg, which appears to be tightly linked to their survival and function in the TME [reviewed in (243)]. Whether combinations with therapeutic approaches targeting Treg or their function result in increased efficacy has not extensively been studied and requires further investigation. Curtin and colleagues reported that anti-CD25 depletion of Treg in combination with intratumoral delivery of an adenoviral vector expressing Fms-like tyrosine kinase 3 ligand and herpes simplex type 1-thymidine kinase inhibited clonal expansion of tumor antigen-specific T cells, T-cell dependent tumor regression, and long-term survival of animals (239), whereas a trend towards improved survival has been reported for the combination of radiotherapy and anti-IDO (244). Particularly, the timing of Treg depletion appears to be of utmost importance. Several of the target molecules (e.g., CD25 and CTLA-4) are not specific to Treg but are upregulated in the course of activation on conventional T cells as well. Similarly, although anti-PD1 treatment may enhance antitumoral immunity, at the same time, it may increase the suppressive activity of Treg (245). Thus, depletion has to be performed prior to the promotion of antitumoral immunity or other treatments, to avoid suppression of the antitumoral effector response (145, 239, 246). Moreover, long-term survival of glioma-bearing mice was only observed when the animals were treated by a combination of systemic and intracranial, but not by systemic anti-CD25 antibody treatment alone (247). Thus, depleting/blocking agents have either to be administered directly to the tumor in the brain or to be able to pass the blood–brain barrier efficiently. Local administration may also prevent uncontrolled inflammation and autoimmune phenomena due to the systemic elimination of Treg and thereby of an important control mechanism of immunity and tolerance.

Evidence for a prominent role of Treg for the efficacy of DCV comes from several studies: Driessen et al. identified a lower Treg frequency in cured rats compared with non-cured rats after DCV in a 9L gliosarcoma model (248). Fong et al. and Prins et al. observed a prolonged survival in patients whose Treg decreased after vaccination with peptide-pulsed DC (67, 93), with higher Treg values before vaccination having been shown by Erhart et al. to be negatively associated with survival after vaccination with lysate-loaded DC (77). Although Batich et al. described long-term survivors after anti-CMVpp6 DCV, they observed increases in Treg that were, however, paralleled by an increase of the CD8^+^:Treg ratio (74). Thus, the absolute numbers or frequencies of Treg by themselves may not be informative enough. Moreover, several studies reported a beneficial effect on survival when DCV was combined with anti-CD25 treatment, particularly when depletion was performed prior to vaccination (106, 227, 249). Thus, depletion of Treg or interference with their activity has the potential to improve the outcome of DCV.

### Myeloid-Derived Suppressor Cells

MDSC are a heterogeneous population of immature myeloid cells with potent immunosuppressive activity. In the TME, they constantly interact with the infiltrating T cells, particularly CTL, and suppress their function (250, 251), thereby supporting tumor growth and progression (252). MDSC are divided into two general subsets: polymorphonuclear (PMN)-MDSC (CD11b+/CD14^−^/CD15+ or CD11b^+^/CD14^−^/CD66b^+^), similar to neutrophils, and monocytic MDSC (CD11b^+^/CD14^+^/HLA-DR^−^/lo//CD15^−^), similar to monocytes (250). However, while they are phenotypically similar to neutrophils and monocytes, they are functionally distinct (253, 254).

MDSC can be detected in cancer patients or during chronic inflammation (250, 251, 255), when persistent low-level stimulation of myelopoiesis results in the development of the immunosuppressive myeloid cells (256). They develop in the bone marrow and traffic into solid tumors, where they accumulate mediated by factors such as GM-CSF, M-CSF, G-CSF, VEGF, IFNγ, IL-6, and IL-4, which are secreted by the tumor cells themselves or other cells of the TME (257, 258).

MDSC, similar to Treg and in part overlapping (see above), use multiple mechanisms of immunosuppression [for review, see (259)], in particular including reactive oxygen species (ROS) and nitric oxide (NO)-dependent pathways. ROS and NO cause apoptosis of immune cells (260) and block entry of CTL into the tumor and responsiveness of T cells to HLA stimulation through the nitration of chemokines and T-cell receptors, respectively (261, 262). MDSC also inhibit extravasation of T cells by downregulating CD44 and CD164 (259) and lymph node re-circling through downregulation of CD62L on naïve T cells *via* expression of ADAM17 (263). Arginase 1 catabolizes the non-essential amino acid arginine and depletes it from the microenvironment. Similarly, there is a depletion of cysteine by consumption and sequestration and of tryptophan due to IDO activity. This lack of amino acids in the TME inhibits proliferation of activated T cells. Like Treg, MDSC can produce immunosuppressive cytokines such as IL-10 and TGF-β and extracellular adenosine through the enzymatic activity of CD39 and CD73. Moreover, they express ligands of immune checkpoint regulatory pathways such as PD-L1, PD-L2, CD155, and galectin-9, which dampen and suppress T-cell responses and may even cause T-cell apoptosis upon interaction with their receptors on T cells (259, 264). Moreover, there is extensive crosstalk between MDSC and Treg, further enhancing immunosuppression (265, 266).

In GBM, MDSC are a major immunosuppressive component of the TME (267). They are also significantly increased in the blood of patients, associated with a higher concentration of the MDSC-specific protein S100A8/9 and arginase activity in the serum (268). More recently, Alban et al. confirmed the presence of MDSC in the blood of GBM patients, while this cell population was completely absent in low-grade glioma patients and healthy individuals (269). In the tumor, MDSC can be found in close proximity to cancer stem cells, and their presence correlates negatively with OS (270). Analogous to Treg, MDSC appear to be recruited to the brain tumor in an early phase and have already been detected in premalignant lesions in a mouse model (271).

Early studies have confirmed the immunosuppressive activity of MDSC in glioma patients and shown that their depletion can restore the disturbed T-cell function (272). Depletion of MDSC with low-dose 5-fluorouracil (5-FU) resulted in prolonged survival in a glioma mouse model (270), and in a clinical study, it has been documented that metronomic capecitabine reduces MDSC, associated with an increase in T-cell infiltrates in the tumors (273). Moreover, their frequency correlates negatively with the response to immunotherapy as reviewed by Stewart and Smyth (274).

Thus, there is evidence for a prominent role of MDSC-mediated immunosuppression in GBM. It has been proposed that targeting MDSC might improve the response to other therapeutic approaches, particularly immunotherapy. The following main targeting strategies are considered: 1) depletion of MDSC, 2) blockage of their migration towards the tumor site, 3) abrogation of their immunosuppressive activity, and 4) pushing them into differentiation towards mature myeloid cells (258). Gao et al. have recently summarized all studies and agents targeting MDSC (275). Therefore, in the following section, only a few examples of the intervention with the immunosuppressive mechanisms of MDSC are presented.

The depletion of MDSC can either be achieved directly by low-dose chemotherapy with, e.g., 5-FU (270), capecitabine (273), or ibudilast (269), or indirectly by promoting their differentiation to either M1 macrophages by docetaxel (276) or towards DC with paclitaxel (277). Full maturation of MDSC can be induced by all-trans retinoic acid [ATRA; (278)]. Blocking of the CSF-1 receptor (CSF-1R) signaling *via* pexidartinib reduced MDSC as well as M2 macrophages (279), and STAT3 inhibitors can reduce the number of MDSC and interfere with their functional activity (271). Furthermore, because MDSC share several mechanisms of immunosuppression with Treg such as the utilization of checkpoint regulatory pathways, the same inhibiting strategies can be used (see *Regulatory T Cells* section).

Evidence for a role of MDSC for efficacy of DCV comes from a study on small cell lung cancer patients, who were vaccinated with p53-loaded DC together with ATRA treatment. DCV alone did not change the frequency of MDSC, which however was reduced twofold by ATRA. Moreover, the combination therapy resulted in a significant increase of specific immune responses (280). In GBM, standard radiochemotherapy has been reported to reduce MDSC in a mouse model (281), but effects have not yet been assessed in humans in detail. Nevertheless, there may be a rationale for depletion of MDSC or interference with their activity together with DCV.

### Tumor-Associated Macrophages

Macrophages display a high plasticity in response to microenvironmental cues, allowing them to acquire distinct phenotypes and perform diverse functions. In general, macrophages are divided into classically activated M1 macrophages and alternatively activated M2 macrophages (282, 283). Differentiation of monocytes to M1 macrophages is induced by GM-CSF and proinflammatory cytokines like IFNγ and TNFα, whereas M2 macrophages differentiate in the presence of M-CSF and anti-inflammatory stimuli (284–288). M2 macrophages can be further subdivided into M2a, M2b, and M2c macrophages (289, 290), with IL-4 and IL-13, immune complexes and TLR agonists, and IL-10, TGF-β, and glucocorticoids representing the major polarizing factors, respectively. Functionally, M1 macrophages are proinflammatory, promoting immunity, wound healing, and tissue regeneration (291, 292). In contrast, M2 macrophages are rather anti-inflammatory; and involvement in wound healing, tissue repair and T_H_2 polarization (293–295), phagocytic and immunomodulatory activity (293, 296–299), and involvement in immunosuppression and angiogenesis (289, 300, 301) have been reported for the M2a, M2b, and M2c subtypes, respectively.

TAM are components of the TME. They frequently exhibit an M2-like phenotype and generally act pro-tumorally, and their presence is associated with poor prognosis. They originate from bone marrow-derived circulating monocytes and accumulate in the tumor due to the presence of M-CSF, GM-CSF, and CCL2 as well as other factors (302, 303). In the tumor, they differentiate into anti-inflammatory M2-like TAM by tumor and TME-derived factors like M-CSF, IL-4, IL-10, and TGF-β. Depending on the local conditions, they polarize towards the M2a, M2b, or M2c subtypes (296, 302, 304–306).

Besides other tumor promoting activities, the anti-inflammatory M2-like TAM promote immunosuppression. They express immunosuppressive cytokines such as TGF-β and IL-10, which not only inhibit T-cell proliferation and function but also contribute together with chemokines, such as CCL20, CCL22, and CXCL12, to an extensive crosstalk with Treg and MDSC, further enhancing immunosuppression (307–314). Moreover, similar to Treg and MDSC, depletion of amino acids (tryptophan and arginine) and expression of ligands of immune checkpoint regulatory pathways contribute to the immunosuppressive activity of TAM (201, 312, 315–320).

In GBM, TAM make up approximately 30%–50% of all cells in the TME (321–323). They are associated with poor prognosis and tumor progression (324–327). Besides infiltrating TAM, in GBM, there is a secondary population of brain-resident monocytic cells, the microglia, which may also be modulated in its activity by the tumor and other cells of the TME (328–331). Bone marrow-derived monocytes are recruited to the brain by cytokines and chemokines such as IL-1β and CCL2 (313, 332), where they mainly differentiate towards anti-inflammatory M2-like macrophages, particularly towards M2c (330, 333). Moreover, the polarization towards M2-like macrophages is an evolving process, which is highly dependent on a hypoxic TME. With increasing hypoxia, the cells polarize more and more towards the M2 subtype (334–336). In line with this observation, there appears to be an increase in M2-like macrophages in recurrent compared with primary tumors, especially in the mesenchymal subtype due to NF-1 deficiency (296, 337).

Due to their importance in promoting tumor progression and immunosuppression, therapeutic targeting of TAM and their function is being attempted. Choi et al. have summarized different molecular targets in preclinical and clinical investigations (338). These include blocking recruitment of TAM into the tumor by targeting chemokines such as CCL2 and CXCL12, which resulted in a reduction in tumor size. Further approaches target the functional characteristics of macrophages, by utilizing small-molecule inhibitors for PI3K, Ras/MAPK signaling, or the IDO pathway, leading, e.g., to reduced IL-10 secretion from M2 macrophages.

A different approach is the reversion of the M2 phenotype to the proinflammatory M1 phenotype by using, e.g., oncolytic virotherapy. Van den Bossche et al. cultured human M2 macrophages with cancer stem cells infected with Delta24-RGD virus and observed a transition towards the M1 phenotype of the cells. Patients treated with this viral particles showed an increase in M1 macrophages in their tumor tissue compared with untreated controls (339). Saha et al. demonstrated that the application of an oncolytic herpes simplex virus (oHSV) expressing IL-12 in combination with antibodies against CTLA-4 and PD-L1 shows a regression of almost all tumors in two GBM mouse models by inducing an effector T-cell influx and an increase in the M1 phenotype of macrophages (340). Thus, local immunostimulatory conditions in the tumor may alter the immunosuppressiveness of the TME. Indeed, DCV in a mouse model revealed a reduction of TAM and MDSC after treatment (341). Moreover, Dammeijer et al. reported that the kinase inhibitor PLX3397 (pexidartinib), targeting CSF-1R signaling, results in a reduction of TAM in a mouse model for malignant mesothelioma but did not influence survival. However, when combined with DCV, survival was increased, which was associated with a reduction of TAM and an increase in effector T-cell infiltration (342). These results suggest that combining DCV with depletion, blocking, or re-polarization of TAM may improve efficacy of the treatment of GBM.

### Immune Checkpoint Regulation

T-cell activation by DC for antitumoral immunity requires the differentiation and expansion of antigen-experienced effector memory T cells (343), with interferon-γ (IFNγ)-producing T_Helper_1 (T_H_1) cells being required for efficient induction of antitumoral effector cytotoxic T cells (121). Antigen-experienced effector memory T cells are characterized by the expression of the surface markers CD45RO and CD69 (and CD103 on tissue resident cells) in the absence of CCR7 (C-C chemokine receptor type 7) and CD62L (343–345). Effector function of these T_H_1 and T_c_1 cells is defined by the expression of the transcription factors (TF) T-bet and Eomes, and the effector potency depends on a delicate balance of these two TFs (346). Highly potent effector T cells present with a T-bet^high^/Eomes^low^ profile. They produce high levels of IFNγ, perforin, and granzyme B (347).

The activity of these effector T cells needs to be tightly regulated in order to prevent excessive immune reactions and uncontrolled inflammation, which may cause destruction of healthy tissue. Therefore, in the course of activation, T cells upregulate immune checkpoint receptors on their surface, including PD-1, CTLA-4, Lag-3, and Tim-3 that upon interaction with their ligands provide a negative feedback to attenuate proliferation and function of the activated T cells, thereby preventing overreactions (348, 349).

In various cancers, including GBM, these immune checkpoint mechanisms, which normally promote self-tolerance and protect against autoimmunity, contribute to tumor immune escape (348–350). Tumor cells or components of the TME such as TAM, Treg, and MDSC express ligands of immune checkpoint receptors, which upon interaction with their receptors on tumor infiltrating T cells cause partial dysfunction of the T cells, a process also referred to as “T-cell exhaustion,” because similarly dysfunctional T cells can be found in chronic infections and after repetitive T-cell stimulation (348, 349, 351). Compared with CD8^+^ T cells generated in response to acute infections, such as an acute CMV infection, exhausted antigen-specific CD8^+^ T cells generated in response to chronic infections or cancer are characterized by reduced proliferation rates, diminished cytotoxicity, and lower cytokine production. Additionally, they express non-transiently checkpoint receptors. T-cell dysfunction has been observed in an early stage of cancer, and it becomes more severe upon tumor progression (352, 353), protecting the tumor cells from the effector mechanisms of the T cells.

In GBM, PD-1/PD-L1 is the best characterized immune checkpoint mechanism. In the majority of tumors, cells of the TME (354) as well as tumor cells express PD-L1 (355), although expression may be restricted to a minor subpopulation of tumor cells only [0%–87%; median 2.8%; (356)]. The respective receptor, PD-1, is expressed on tumor-infiltrating CD4^+^ and CD8^+^ T cells (355). Expression on the tumor-infiltrating T cells is higher than on their counterparts in blood (346, 357), and they are functionally impaired (346, 358). Besides PD-1, expression of Tim-3, Lag-3, and CTLA-4 has also been observed on infiltrating T cells in GBM (359).

The dysfunctional state of the T cells may not be permanent but appears to have to be maintained by receptor–ligand interactions, in contrast to T-cell senescence, which is not reversible (353, 360). When the respective immune checkpoint receptors are blocked, e.g., by receptor or ligand-specific monoclonal antibodies, T cells can be reinvigorated: their function and proliferation are restored (359, 361). Indeed, in many tumor entities, application of monoclonal antibodies blocking immune checkpoint receptors or their ligands has resulted in a survival benefit for the patients (361).

In mouse models of GBM, blocking of CTLA-4, PD-1/PD-L1, and TIGIT has been shown to result in increased survival, frequently associated with depletion of immunosuppressive cells and an influx of effector T cells into the tumor (222, 228–231). Thus, there appears to be an intrinsic antitumoral immune response in cancer patients, which can be enhanced by interference with the immune checkpoint pathways. Although this type of immunotherapy results in durable responses in many tumors, this is only true for a fraction of patients (20%–50%, depending on the cancer type), and therapy is associated with severe immune-related adverse events (362). In contrast to many other tumors, however, in GBM, interference with immune checkpoint pathways has not been successful. No survival benefit has been reported so far in several clinical trials (363–367), except for one of three trials applying an anti-PD-1 monoclonal antibody in a neoadjuvant setting (368–370). The reason for the therapeutic failure of immune checkpoint interference in GBM is currently unknown. Whether other checkpoint regulators or possibly other immune escape mechanisms play a more prominent role in GBM than in other tumors and therefore the loss of PD-1 signaling due to blocking is irrelevant remains to be determined. However, reinvigoration of exhausted T cells may also have limitations in GBM. It depends on how terminally differentiated/exhausted T cells are. The function of exhausted T cells showing a T-bet^low^/Eomes^high^ TF expression and a concomitant expression of multiple immune checkpoint receptors cannot be restored (346). It is further essential to distinguish between progenitor exhausted T cells, which express the TF TCF1 and the TCF1^−^/Tim-3^+^ terminally exhausted T cells, which co-express the TF TOX, vital for their persistence in the tumor environment with chronic antigen stimulation, with the later population not being re-invigoratable, thus not responding to checkpoint blockade (352, 353, 371). It has been suggested that immune checkpoint inhibition improves OS by overcoming the exhaustion state of tumor-infiltrating T cells resulting in an increased effector response, but functional proof about that is still missing up to date. Although several studies have described an increase in TCF1^+^ tumor-infiltrating T cells, it is unclear whether they are re-invigorated from the exhausted cells, represent new “non-exhausted” or “non-terminally differentiated” clonotypes, or are recruited from the periphery following immune checkpoint inhibition.

Furthermore, immune checkpoint interference can only enhance but not induce antitumoral responses. The low mutational load [(94); for review, see (95)] and the low frequency [20%–30% (50, 83)] of preexisting antitumoral T-cell responses in GBM patients may therefore limit efficacy of immune checkpoint interference as well. In agreement with this, PD-1 or PD-L1 blockage combined with DCV in mouse models resulted in CD8^+^ T cell-dependent long-term survival, which was not observed with the respective monotherapies (372, 373). Similar results have been obtained by Wang et al., who vaccinated GBM patients with personalized TAA-pulsed DC combined with low-dose cyclophosphamide, poly(IC), imiquimod, and anti-PD-1 antibody, which induced antigen-specific CD4^+^ and CD8^+^ T-cell responses, which were associated with a favorable outcome when compared with the respective monotherapies (86). Moreover, Jan et al. and Yao et al. reported for DCV that a lower PD-1^+^:CD8^+^ ratio in TIL as well as in blood lymphocytes is associated with longer survival (78, 81), and Fong et al. described an association of decreased CTLA-4 expression with survival after DCV (93). Thus, there appears to be a rationale for combining DCV and blocking of immune checkpoint regulatory pathways to increase efficacy. DCV itself might be able to trigger the expansion of the above-described TCF1^+^/TOX^−^ neoantigen-specific T cells from the periphery or even the tumor site and hence enhance tumor killing and survival.

Overall, targeting any one of the three immunosuppressive cell populations in the TME of GBM as well as the immune checkpoint regulatory pathways (**Figure 2**) appears to represent a promising approach by itself, but in particular in combination with DCV.

## Conclusion

Even after more than 10 years of DCV in GBM and after more than 1,000 patients having been vaccinated, it is still difficult to draw conclusions as to the efficacy of DCV. However, there are promising results urging to further develop it as a therapeutic tool, which will require not only optimizing the DC vaccines in respect to target antigen selection, preparation of the cells, and integration of DCV into other treatment regimens but also dosing and scheduling of the vaccination(s). Future vaccination strategies will have also to take into account immunosuppression in GBM and the means to overcome it, which are now becoming increasingly available, to improve efficacy in GBM patients.

## Author Contributions

Both authors contributed equally to the article and approved the submitted version.

## Funding

This study was supported by a grant from the Federal Ministry of Education and Research (BMBF; grant #01KG1242).

## Conflict of Interest

The authors declare that the research was conducted in the absence of any commercial or financial relationships that could be construed as a potential conflict of interest.

## Publisher’s Note

All claims expressed in this article are solely those of the authors and do not necessarily represent those of their affiliated organizations, or those of the publisher, the editors and the reviewers. Any product that may be evaluated in this article, or claim that may be made by its manufacturer, is not guaranteed or endorsed by the publisher.
